# Estimating missing values in China’s official socioeconomic statistics using progressive spatiotemporal Bayesian hierarchical modeling

**DOI:** 10.1038/s41598-018-28322-z

**Published:** 2018-07-03

**Authors:** Chao Song, Xiu Yang, Xun Shi, Yanchen Bo, Jinfeng Wang

**Affiliations:** 1 School of Geoscience and Technology, Southwest Petroleum University, Chengdu, Sichuan 610500 China; 2 Department of Geography, Dartmouth College, Hanover, New Hampshire 03755 USA; 3 China Science and Technology Exchange Center, Division of Policy Study, Beijing, 100045 China; 4 State Key Laboratory of Remote Sensing Science, Faculty of Geographical Science, Beijing Normal University, Beijing, 100875 China; 5 LREIS, Institute of Geographic Sciences and Natural Resources Research, Chinese Academy of Sciences, Beijing, 100101 China

## Abstract

Due to a large number of missing values, both spatially and temporally, China has not published a complete official socioeconomic statistics dataset at the county level, which is the country’s basic scale of official statistics data collection. We developed a procedure to impute the missing values under the Bayesian hierarchical modeling framework. The procedure incorporates two novelties. First, it takes into account spatial autocorrelations and temporal trends for those easier-to-impute variables with small missing percentages. Second, it further uses the first-step complete variables as covariate information to improve the modeling of more-difficult-to-impute variables with large missing percentages. We applied this progressive spatiotemporal (PST) method to China’s official socioeconomic statistics during 2002–2011 and compared it with four other widely used imputation methods, including k-nearest neighbors (kNN), expectation maximum (EM), singular value decomposition (SVD) and random forest (RF). The results show that the PST method outperforms these methods, thus proving the effects of sophisticatedly incorporating the additional spatial and temporal information and progressively utilizing the covariate information. This study has an outcome that allows China to construct a complete socioeconomic dataset and establishes a methodology that can be generally useful for estimating missing values in large spatiotemporal datasets.

## Introduction

Official socioeconomic statistics data are fundamental to policy-making and multi-disciplinary research^1^. In China, some official socioeconomic databases are openly available from different sources, such as the China Data Center (http://chinadatacenter.org/default.aspx), the Thematic Database for the Human-Earth System (http://www.data.ac.cn/index.asp) and special research programs^2^. However, a common problem with these databases is that they are all at the coarse provincial level. The county is the basic data collection unit for the official statistics in China, while the published county-level socioeconomic yearbooks have a considerable amount of missing data. Thus far, the country has not published any socioeconomic statistics databases that completely cover the entire country with data at the county level for an extended period^3,4^, due to the insufficient survey and data collection infrastructure, especially in some remote areas. This county-level missing data problem has seriously limited the wide use of China’s socioeconomic data.

Various methods for estimating missing values have been applied to official statistics data^5,6^. The traditional design-based methods, such as the James-Stein estimator^7^ and the generalized regression estimator^8^, are commonly used, but they require sufficient samples, which are hard to acquire in many regions. To avoid the challenge of collecting samples, the auxiliary information from other related variables^6^ has been used to impute the missing data for the official statistics in different countries^9–11^. For instance, a demographic study with census data in the US utilized the covariate information in each area for estimating missing values^11^, and a cultural participation study in Australia estimated missing data with an additional synthetic database^9^. However, useful auxiliary information is not always available in practice, similar to samples^12^. Particularly in China, neither samples nor auxiliary data are available for the socioeconomic dataset on the spatiotemporal scales.

When neither samples nor auxiliary data are available, model-based imputation methods have been proposed, such as k-nearest neighbors (kNN)^13^, expectation maximum (EM)^14^, singular value decomposition (SVD)^15^, and random forest (RF)^16^. For each record whose value is missing, kNN finds its k nearest neighbors whose values are available using the Euclidean metric and imputes the missing value by averaging the values of the neighbors^13,17^. EM assumes a distribution for the partially missing data and bases inferences on the likelihood of that distribution^14^. SVD initializes all missing records with zeros and then estimate them as a linear combination of the k most significant eigen-variables until it reaches a certain convergence threshold^13,15^. RF imputes data by regressing each variable in turn against all other variables and then predicting missing data for the dependent variable using the fitted forest^16^. However, none of these methods have taken into account information about spatial and temporal structures in the estimation of missing values.

Spatial agglomeration is a common socioeconomic phenomenon^18^. Thus large-scale official statistics data usually have spatial structures, particularly spatial autocorrelation^19^. Moreover, temporal autocorrelation, in which observations that are temporally close to each other tend to be similar, is also likely to be inherent in official statistics data^20,21^. On the one hand, information about spatial and/or temporal structures can be utilized for estimating missing values, especially when other information, such as that from samples and auxiliary data, is unavailable. In addition, for a spatiotemporal dataset, an imputation model that cannot fully capture the spatial and/or temporal structures in the data may introduce bias into the results, thus leading to low accuracy and high uncertainty^22^. Unfortunately, the four widely used model-based imputation methods described above are not designed to incorporate either spatial or temporal autocorrelation effects.

Under this situation, spatial statistical models can be applied to estimate missing values by accounting for spatially correlated information as spatial components in the model^23^. For example, Bihrmann, K. *et al*. implemented a logistic regression model with a spatially structured random component to impute missing data on Salmonella Dublin in Danish cattle herds^24^. Baker, J. *et al*. used a Bayesian model with the spatial intrinsic conditional autoregressive prior to impute missing data in health studies^25^. Staubach, C *et al*. used a beta-binomial model incorporating spatially structured and unstructured random effects to complete disease prevalence data^26^. However, the spatial information has not been widely taken into account in estimating missing values for official statistics data.

In addition, most spatial models for estimating missing data focus on a snapshot situation and neglect the temporal autocorrelations in the data^24–26^. Furthermore, the spatial and temporal structures can have interactivity, which may not be fully captured if the model only considers the two effects separately^27^. To address these problems, spatiotemporal models are usually formulated within the Bayesian hierarchical modeling (BHM) framework to account for spatial structures, temporal structures and their respective space-time interactions^28^. BHM is a powerful analytical technique for building spatiotemporal statistical models^29^, as the information provided by neighboring regions and time trends can be naturally represented as priors and it gives robust posterior estimates^30^. BHM-based spatiotemporal models have been found in many applications^31–33^, but have not been to missing data estimations of official statistics.

To estimate missing values for China’s socioeconomic official statistics data, for which the problem that neither samples nor auxiliary information are available is frequently encountered, we developed a spatiotemporal modeling procedure under the BHM framework that incorporates spatial autocorrelation, temporal correlation, and space-time interactions as the primary information sources. In addition, this modeling procedure is progressive since it contains two steps. It first imputes those easier-to-impute variables that have only small percentages of missing values, for which models considering only spatial and temporal information can achieve a decent estimation quality. It then uses the estimation results of these easier-to-impute variables as covariate information, along with the spatiotemporal multivariate regression model, to impute those more-difficult-to-impute variables that have large percentages of missing values.

We applied this progressive spatiotemporal (PST) procedure to the estimation of county-level missing data in China’s official socioeconomic statistics from 2002 to 2011. We evaluated different types of spatiotemporal models in order to select an optimal implementation for the PST method. We also evaluated PST’s sensitivity to a change of the missing data percentages and created spatial uncertainty maps. As a comparison, we also applied four other imputation methods, including kNN, EM, SVD and RF to the Chinese dataset.

To our best knowledge, no previous works on missing data estimations of official statistics have constructed BHM-based spatiotemporal models that comprehensively incorporate spatial, temporal, and covariate effects and perform modeling in a progressive way. In the area of official statistics, studies on such a great scale and such a large dataset that cover all of China are rare.

## Methods

### Study area and data

The socioeconomic data that we used in this study are from three series of official statistics yearbooks published by the National Bureau of Statistics of China (http://www.stats.gov.cn/english/), including the China County Statistical Yearbook, the China Statistical Yearbook for Regional Economy, and the China City Statistical Yearbook^34^. Data of counties (suburban/rural areas) in China are from the former two yearbooks, in which the statistical variables complement each other. In China’s administrative division system, a city can contain a number of county-level units called municipal districts. The data of municipal districts of cities are from the latter yearbook, which contains the complete set of statistical variables. We conducted logarithmic transformation of each socioeconomic variable to approximate a normal distribution^1,35,36^ in order to mitigate the impact of extreme values, and to make the effective relationships non-linear while still preserving the linearity of the model^37^.

The situations of missing data are different across statistical variables and yearbooks, and are generally more serious in the China County Statistical Yearbook and the China Statistical Yearbook for Regional Economy. If we use the county-year as a unit, in these two series of yearbooks, across different variables the minimum missing percentage is 28.69% during the 10-year period of 2002 to 2011, the maximum is 36.08%, and the mean is 30.98%. On the other hand, the China City Statistical Yearbook is almost complete, but it only covers urban areas. We combine the data from these three series into an integrated dataset. The dataset covers a total of 20 socioeconomic variables for 2,310 county-level areal units in China spanning a 10-year period from 2002 to 2011.

**Figure 1:**
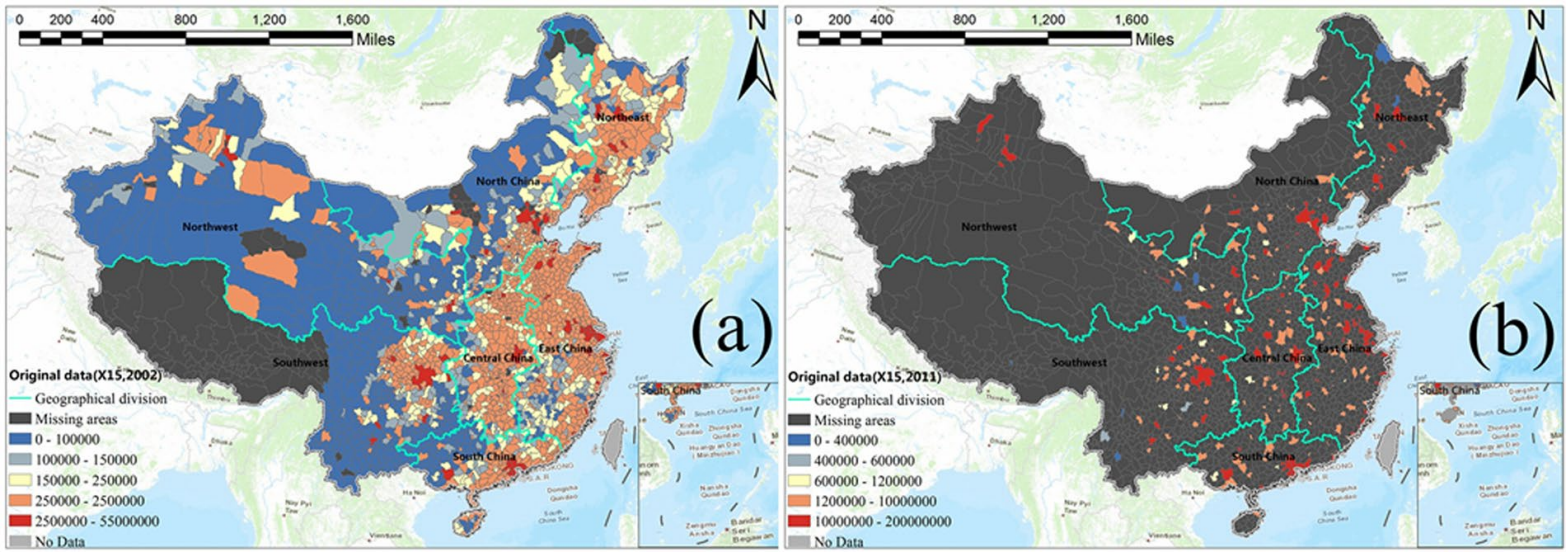
Study area and the missing data maps of GDP (variable X15) in the years 2002 (**a**) and 2011 (**b**).

For each of the 20 socioeconomic variables in our integrated dataset, we calculated its overall percentage of county-years with missing data during the 10-year period. As shown in Table 1, the overall missing-data percentages of the last six variables (X15 to X20) are much larger than those of the first 14 variables (X1 to X14). We also calculated for each variable the maximum yearly percentage of counties with missing data during the 10 years, based on which we defined that if the percentage is <15%, then the quality of the data for that variable in that year is acceptable, e.g., Fig. 1(a). If it is >85%, the quality is unacceptable, and we named this year a big year of data missing for that variable, e.g., Fig. 1(b). It turned out that a big year of data missing only appears with variables X15-X20. Figure S1 in the additional document provides the detailed information about missing data for all counties in an example year. Based on whether a variable has at least one big year of data missing, we divided the dataset into two parts the first 14 variables (X1 to X14), which have no big year of data missing, and the last six variables (X15 to X20), which have big years of data missing. These two parts were separately used in the two steps of modeling.

**Table 1:** Missing data situations of 20 socioeconomic variables.

Abbreviation	Socioeconomic variable	Unit	Overall missing percentage	Max missing percentage	Number of big missing data years
X1	Land area	km^2^	2.25%	5.54%	0
X2	Total population	person	2.19%	5.50%	0
X3	Employees at the end of the year	number	2.40%	5.58%	0
X4	Local telephone users at the end of the year	person	2.91%	6.02%	0
X5	Local general budget revenue	million	2.42%	5.58%	0
X6	Local government budgetary expenditures	million	2.37%	5.63%	0
X7	Savings deposits of urban and rural residents	million	2.86%	6.02%	0
X8	Loan balance of financial institutions	million	2.65%	5.84%	0
X9	Total retail sales of social consumer goods	yuan	4.58%	7.23%	0
X10	Above-scale total industrial output value	million	6.47%	14.11%	0
X11	Social fixed asset investments	million	3.12%	6.06%	0
X12	Middle and high school students	person	2.34%	5.58%	0
X13	Primary school students	person	2.25%	5.45%	0
X14	Number of hospital beds	number	2.37%	5.50%	0
X15	Regional GDP	million	12.39%	87.66%	1
X16	First industry output	million	20.90%	87.62%	2
X17	Second industry output	million	20.88%	87.62%	2
X18	Tertiary industry output	million	29.51%	87.66%	3
X19	GDP per capita	yuan/person	38.57%	88.01%	4
X20	Staff and workers in Urban Units	person	15.34%	87.66%	1

(We use X1 to X20 to refer to the 20 variables. The missing percentage is the ratio of the total number of the county-years with missing data for a variable to the total number of county-years during the 10-year period).

### Experimental design

**Figure 2:**
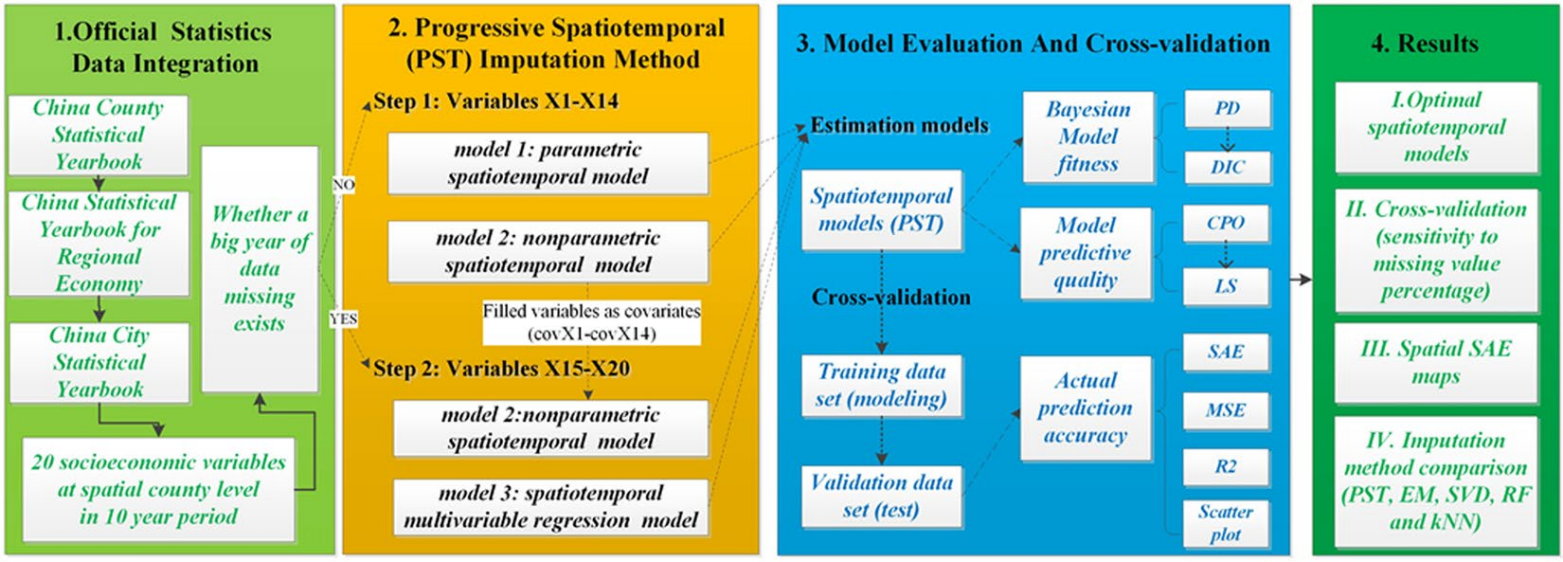
Experiment’s overall design flow chart.

The overall design of analysis is illustrated in Fig. 2. Before modeling, we started with a Moran’s I test on the spatial autocorrelation of each target socioeconomic variable in each year (supplementary file S3). Because the counties of China vary greatly in size, where some are very large and some are very small, we chose to use contiguity rather than distance to represent the spatial relationship in measuring the spatial structure. We found that for all socioeconomic variables in each year, the Z-score was positive and significant (>2.58), which indicates that all the variables have significant spatial autocorrelations and that it is reasonable to utilize the spatial autocorrelation information for imputing missing data.

The PST modeling process contains two general steps. In the first step, we derived information from the spatial and temporal structures in the existing data. We used the spatiotemporal models that take into account of the random effects of spatial, temporal, and their interactive imformation to estimate the missing values for variables X1 to X14, whose missing percentages are small, with no auxiliary covariates or samples involved (as they were not available). The second step worked for variables X15 to X20, whose missing percentages are large. The second step used multivariable regression modeling because we had the covariates from the first step as the independent variables.

In each of the two steps, we built two alternative statistical models. In step 1, we built two spatiotemporal models, one parametric^38^ and one nonparametric^27^ (herein referred to as Model 1 and Model 2, respectively). They have the common components of spatial effects, but Model 1 uses the linear time prior, whereas Model 2 uses the nonlinear time prior for the temporal components and the space-time interaction components. With Models 1 and 2, we intended to discover which type of spatiotemporal model is more suitable for our data, and we chose the optimal one between the two for the next step. In step 2, we constructed spatiotemporal multivariable regression models (herein referred to as Model 3). Compared with Model 2, Model 3 incorporates additional covariate information (the 14 variables imputed in step 1). Model 3 will demonstrate the usefulness of the new imputed covariates in estimating other variables.

After building the models, we used a variety of methods for evaluation and validation. First, we evaluated the two pairs of alternative spatiotemporal models (Models 1 vs. 2 and Models 2 vs. 3) regarding the Bayesian model fitness using the deviance information criterion (DIC) and the predictive quality using the conditional predictive ordinate (CPO). This first step of the evaluation was based on the entire dataset and selected an optimal spatiotemporal model for the PST imputation. Second, we ran a cross-validation to evaluate the predictive performance of PST and the model’s sensitivity to change of a missing data percentage. Specifically, we randomly sampled 10%, 20%, and 30% from the existing data to create three test sets, and used the rest of the data as the training sets.We further obtained the spatial uncertainty maps to evaluate the local prediction errors of the spatiotemporal models applied in the PST method. Third, we compared the proposed PST method with four other widely used imputation methods, including kNN, SVD, EM, and RF. We applied cross-validation (10% random samples) to test the actual accuracy of these imputation methods, and implemented the four methods using R.

### Statistical methods

#### Progressive spatiotemporal (PST) modeling

Spatially, we denote the 2,310 county-level areal units as *i* = *1, …, I* (*I* = 2310). Temporally, we denote the 10 years as *t* = *1, …, T* (*T* = 10). Let y_it_ denote the values of a socioeconomic variable in area *i* and year *t*. All of our models assume a log-normal likelihood prior distribution. The structured additive linear predictor ${\eta }_{it}=\,\mathrm{log}({y}_{it})$ will be decomposed additively into components of space, time, or both. As aforementioned in the Experimental Design section, we constructed three different models. The details are described in this section.

Parametric spatiotemporal model (Model 1)^38^:

1 $${\eta }_{it}=\alpha +{\mu }_{i}+{\nu }_{i}+(\beta +{\delta }_{i})\times t$$

In the linear predictor ${\eta }_{it}$, α quantifies the intercept fixed effect, and *μ*_*i*_ and *v*_*i*_ are the spatial components that represent two random effects. The term *v*_*i*_ assumes a Gaussian exchangeable prior to the model unstructured heterogeneity, which is formalized as ${\nu }_{i} \sim N(0,{\delta }_{\nu }^{2})$, and *μ*_*i*_ assumes an intrinsic conditional autoregressive (CAR) prior for the spatially structured variability.

The spatial components include two effects: one assuming a Gaussian exchangeable prior to model the unstructured heterogeneity, which is ${\nu }_{i} \sim N(0,{\delta }_{\nu }^{2})$, and the other assuming an intrinsic conditional autoregressive (CAR) prior for the spatially structured variability^39^, which is:

2 $${\mu }_{i}|{\mu }_{j\ne i} \sim N(\frac{1}{{m}_{i}}{\sum }_{i \sim j}{\mu }_{i},\frac{{\sigma }^{2}}{{m}_{i}})$$

where *i ~j* indicates that areas *i* and *j* are neighbors, *m*_*i*_ is the number of areas that share boundaries with the *i-th* area and ${\sigma }^{2}$ is the variance component. The spatial dependence in ${\mu }_{i}$ assumes the CAR prior that extends the well-known Besag model^39^, with a Gaussian distribution, which implies that each ${\mu }_{i}$ is conditional on the neighbor ${\mu }_{j}$ with the variance dependent on the number of neighboring counties *m*_*i*_ of county *i*. The structured spatial effect is considered as the spatial autocorrelation information that is borrowed from nearby neighbors, and the unstructured spatial effects are seen as the spatial heterogeneity characteristics in a specific area. Model 1 also includes the linear effect *β*, which represents the main temporal trend, and a differential temporal trend *𝛿*_*i*_, which represents the area-specific time variation (the differential time trend for each region).

Nonparametric spatiotemporal model (Model 2): As an alternative to the assumption of a linear time trend in Model 1, Model 2 implements a general dynamic nonparametric time trend, which is considered more realistic. It adopts a random walk model for the main temporal trend and the corresponding spatiotemporal interaction term. The linear predictor of a nonparametric spatiotemporal model can be written as^27^

3 $${\eta }_{it}=\alpha +{\mu }_{i}+{\nu }_{i}+{\gamma }_{t}+{\varphi }_{t}+{\delta }_{it}$$

where ${\mu }_{i}$ and ${\nu }_{i}$ represent the spatial main effects, which are the same as in Model 1; ${\gamma }_{t}$ and ${\varphi }_{t}$ represent the temporal main effects; and ${\delta }_{it}$ represents the space-time interactions. The term ${\varphi }_{t}$ represents the unstructured time effect and is specified by using an independent mean-zero normal prior with unknown variance ${\sigma }_{\varphi }^{2}$. The term ${\gamma }_{t}$ represents the structured time effect and is modeled dynamically through a neighboring structure. We used the random walk (RW) dynamic model as a prior for the structured time effect, with its prior density π as follows^28^:

4 $$\pi ({\gamma }_{t}|{\sigma }_{\gamma }^{2})\propto \exp (-\frac{1}{2{\sigma }_{\gamma }^{2}}\sum _{t=2}^{T}{({\gamma }_{t}-{\gamma }_{t-1})}^{2})$$

In the time-space interaction term ${\delta }_{it}$, *i* = *1,…,I* is the space index and *t* = *1,…,T* is the time index. The specification of the prior on ${\delta }_{it}$ depends on the spatial and temporal main effects, which are assumed to interact. Assuming that the spatial main effect ${\nu }_{i}$ and the temporal main effect ${\gamma }_{t}$ interact with each other, each spatial unit ${\delta }_{i}=({\delta }_{i1},{\delta }_{i2},\,\mathrm{..}.,\,{\delta }_{iT})^{\prime} ,i=1,\,\mathrm{..}.\,I$ follows a random walk, and the prior on ${\delta }_{it}$ is thereby written as follows^27^:

5 $$p(\delta |{\kappa }_{\delta })\propto \exp \{-\frac{{\kappa }_{\delta }}{2}\sum _{i=1}^{m}{\sum _{t=2}^{T}({\delta }_{it}-{\delta }_{i,t-1})}^{2}\}$$

where ${\kappa }_{\delta }$ is the precision factor, which is the reciprocal of variance ${\sigma }_{\delta }^{2}$. The space-time interactions ${\delta }_{it}$ are considered as unobserved covariates for each unit (*i,t*) that have structures in time and space. Such a specfication is suitable when temporal trends are different among counties but the spatial trends are stable. With ${\delta }_{it}$, Model 2 can take into account not only of the spatial heterogeneity of each county but also the temporal variation of each county across ten years for the missing data imputation.

Spatiotemporal multivariable regression model (Model 3): When covariate information (observed and related variables) is available for imputing missing values, a traditional multivariable regression model can be easily specified as ${\eta }_{it}=\alpha +\sum _{k}{\beta }_{k}{X}_{itk}$, where α quantifies the intercept, *X*_*k*_ is the *k-th* covariate, and ${\beta }_{k}$ are the coefficients^23^. Combining it with Model 2, we build Model 3 as follows:

6 $${\eta }_{it}=\alpha +\sum _{k}{\beta }_{k}{X}_{itk}+{\mu }_{i}+{\nu }_{i}+{\gamma }_{t}+{\varphi }_{t}+{\delta }_{it}$$

where the specifications of these spatial and temporal random effects are the same as in Model 2. With this model, the imputation can comprehensively incorporate the related covariates, spatial effects, temporal effects, and space-time interactions.

### Bayesian hierarchical modeling framework

Bayesian hierarchical modeling (BHM) is a statistical process that works on multiple levels to estimate the parameters of posterior distributions using the Bayesian method^40^. It has demonstrated the advantage of being able to impute missing data in a relatively straightforward way^28^. By applying BHM to spatiotemporal modeling, we implemented the prediction models in this study with three levels, namely, the data distribution, the spatiotemporal process, and the parameter, where each level can also contain a number of sub-levels. We employed the log-normal likelihood model for the data distribution and combined different sub-models (the CAR and RW models) to form a hierarchical model for the spatiotemporal process to incorporate the random effects of the spatial structures, temporal structures, and space-time interaction. For the parameter level, we used the inverse gamma distribution as the priors for all unknown variance parameters. This non-informative prior specification for the parameters and their variance components allows the observed data to have the greatest influence on posterior distributions without being greatly influenced by the choice of the prior^41^. The BHM-based PST models presented in this study were solved using the Integrated Nested Laplace Approximation (INLA) approach in the R software^42^. A major advantage of INLA is that it returns accurate parameter estimates in a relatively short computational time^30^. The R-INLA package can be directly downloaded from http://www.r-inla.org/. The core codes for fitting spatiotemporal models have been openly published in a few studies^28,30,41^.

### Model evaluation and validation

1. Bayesian model fitness
  The deviance information criterion (DIC) is a well-known Bayesian model comparison criterion, which is defined as^43^
  7 $${\rm{DIC}}=\overline{D}+{p}_{D}$$
  where $\bar{D}$ is the mean of the model posterior deviance and ${p}_{D}$ is the effective number of parameters. The greater the value of *p*_*D*_ is, the higher the complexity of the model. The greater the mean deviance values are, the greater the error of the representative model. Models with smaller DICs are better supported by the data.
2. Bayesian model predictive quality
  The conditional predictive ordinate (CPO) is defined as a cross-validated predictive density at a given observation and can be used to compute predictive measures^44^. For continuous distributions, it is defined as
  8 $$CP{O}_{i}=p({y}_{i}^{\ast }|{y}_{f})$$
  where ${y}_{i}^{\ast }$ is the predicted value and ${y}_{f}$ is a sample of observations *y*, which is used to fit the model and to estimate the posterior distribution of the parameters. In practice, the cross-validated logarithmic score (LS) computed from the CPO is used to evaluate the model’s predictive quality. A smaller LS indicates a better prediction of the model. The LS is calculated as
  9 $$LS=-\frac{1}{n}\sum _{i=1}^{n}\mathrm{log}(CP{O}_{i})$$
3. Actual prediction accuracy

To compare different imputation models with cross-validation, we used three indices to measure the actual prediction accuracy, namely, the standardized allocation error (SAE)^11^, the mean square error (MSE) and the coefficient of determination (R^2^)^21,35^. All these indices compare the model-predicted values with observed values. The SAE, MSE, and R^2^ are calculated as follows:

10 $$SAE=\frac{\sum _{i=1}^{n}|{y}_{i}-{y}_{i}^{\ast }|}{\sum _{i=1}^{n}{y}_{i}},\,MSE=\frac{1}{n}\sum _{i=1}^{n}{({y}_{i}-{y}_{i}^{\ast })}^{2},\,{\rm{and}}\,{R}^{2}=1-\frac{\sum _{i=1}^{n}{({y}_{i}-{y}_{i}^{\ast })}^{2}}{\sum _{i=1}^{n}{({y}_{i}-\bar{y})}^{2}},$$

where ${y}_{i}$ is the observed value, ${y}_{i}^{\ast }$ is the predicted value, $\bar{y}$ is the mean of the observed values and *n* is the number of validation samples.

The SAE is a relative error index that is convenient for comparison between alternative models and has been well adopted in the official statistics field to compare various estimation methods^11^. An SAE value close to 0 indicates a good fit between the actual and estimated distributions. In addition, we could calculate the localized SAE for each spatiotemporal unit with $SA{E}_{ij}=\frac{|{y}_{ij}-{y}_{ij}^{\ast }|}{{y}_{ij}}$.

The MSE is an absolute error index. A smaller MSE indicates a better prediction of a model. The R^2^ is an index for assessing the agreement between observed and estimated values, with the value ranging from 0 for complete disagreement to 1 for perfect agreement. Scatterplots were created to compare the observed values and estimated values in the cross-validation^1,21^.

### Data availability

The three governmental yearbook series, which provide the original data for this study, are available from the National Bureau of Statistics of China (http://www.stats.gov.cn/english/). The new datasets generated during the current study are not publicly available due to the limitation of the copyright of the governmental data source but are available from the corresponding authors upon a reasonable request with reference to this paper and a signed confidentiality agreement.

## Results

### Optimal spatiotemporal models

Table 2 lists the evaluation results for the two pairs of alternative spatiotemporal models. For the first 14 variables, between Model 1 and Model 2, the latter has larger *pD* values, which indicate that Model 2 is more complex, apparently because it incorporates a spatiotemporal interaction term that is not a part of Model 1. This higher complexity was beneficial, as it led to lower DIC values, thus indicating a better fit to the data. The higher quality of Model 2 is further confirmed by the lower LS values that represent a better predictive ability.

**Table 2:** Bayesian models’ evaluated results of 20 variables with the alternative spatiotemporal models (M1: parametric spatiotemporal model; M2: nonparametric spatiotemporal model; M3: spatiotemporal multivariable regression model).

Variable	Model	*p*_*D*_	DIC	LS
X1	M1	126.69	125663.17	2.72
	M2	6176.37	−14111.29	−0.45
X2	M1	1835.48	96930.00	2.15
	M2	5543.50	−12130.76	−0.41
X3	M1	3929.79	10166.79	0.18
	M2	8343.87	6623.99	0.10
X4	M1	3820.75	19380.00	0.42
	M2	9028.92	10103.58	0.22
X5	M1	4034.30	19129.21	0.41
	M2	14040.63	2708.83	0.15
X6	M1	3644.52	12854.78	0.26
	M2	8941.07	5934.85	0.12
X7	M1	2103.73	92887.11	2.07
	M2	8027.79	6469.70	0.11
X8	M1	2110.40	93380.78	2.08
	M2	11798.02	5849.11	0.17
X9	M1	1909.06	98419.80	2.24
	M2	14893.63	−1767.84	0.12
X10	M1	4234.27	25353.06	0.60
	M2	14215.22	9917.30	0.42
X11	M1	4079.19	39651.11	0.88
	M2	11832.53	27789.02	0.69
X12	M1	4247.52	3280.72	0.01
	M2	11932.56	−4713.48	−0.12
X13	M1	4072.09	−3645.90	−0.16
	M2	9404.30	−8151.90	−0.27
X14	M1	3946.53	3705.55	0.05
	M2	8774.90	−1100.59	−0.06
X15*	M2	4690.08	29812.04	0.75
	M3	5254.68	28573.29	0.73
X16*	M2	7528.36	32827.42	1.01
	M3	7616.29	31564.11	0.97
X17*	M2	7244.71	38198.39	1.14
	M3	7120.46	37171.88	1.10
X18*	M2	5616.22	21887.47	0.72
	M3	5488.50	20870.88	0.69
X19*	M2	5432.17	2872.51	0.12
	M3	5388.29	2837.99	0.11
X20*	M2	3598.56	33249.81	0.87
	M3	3723.70	32603.66	0.85

^*^Variables belong to the second-step imputation modeling of the PST method.

To select the covariates from the first 14 variables to assist with the prediction of the last six variables, we first assessed the multicollinearity to select the variables whose variance inflation factor (VIF) <5. We then used the forward stepwise regression method to further select those variables that have a significant association (sig <0.05) with the target variable for modeling. The variables selected in this way were considered to have spatial and temporal structures similar to those of the last six variables (see supplementary file section S2 for details) and could be used in Model 3.

Because Model 3 included additional covariate terms, it has a higher complexity than Model 2, as indicated by the *P*_*D*_ values in Table 2. This higher complexity brought better model fitness (lower DIC) and predictive ability (lower LS) to Model 3.

The comparison between Models 1 and 2 indicates the usefulness and necessity of including the main time trend and the space-time interaction. The comparison between Models 2 and 3 demonstrates the effectiveness of the proposed progressive modeling process. That is, easier-to-impute variables (variables with small percentages of missing values) can be helpful in the imputation of those more-difficult-to-impute variables (variables with large percentages of missing values). Through the two step experiment, we selected the nonparametric spatiotemporal model (Model 2) and the spatiotemporal multivariable regression model (Model 3) as the final models for the PST method to perform the imputation for our integrated dataset of China’s official statistics.

### Cross-validation and sensitivity to missing value percentage

**Figure 3:**
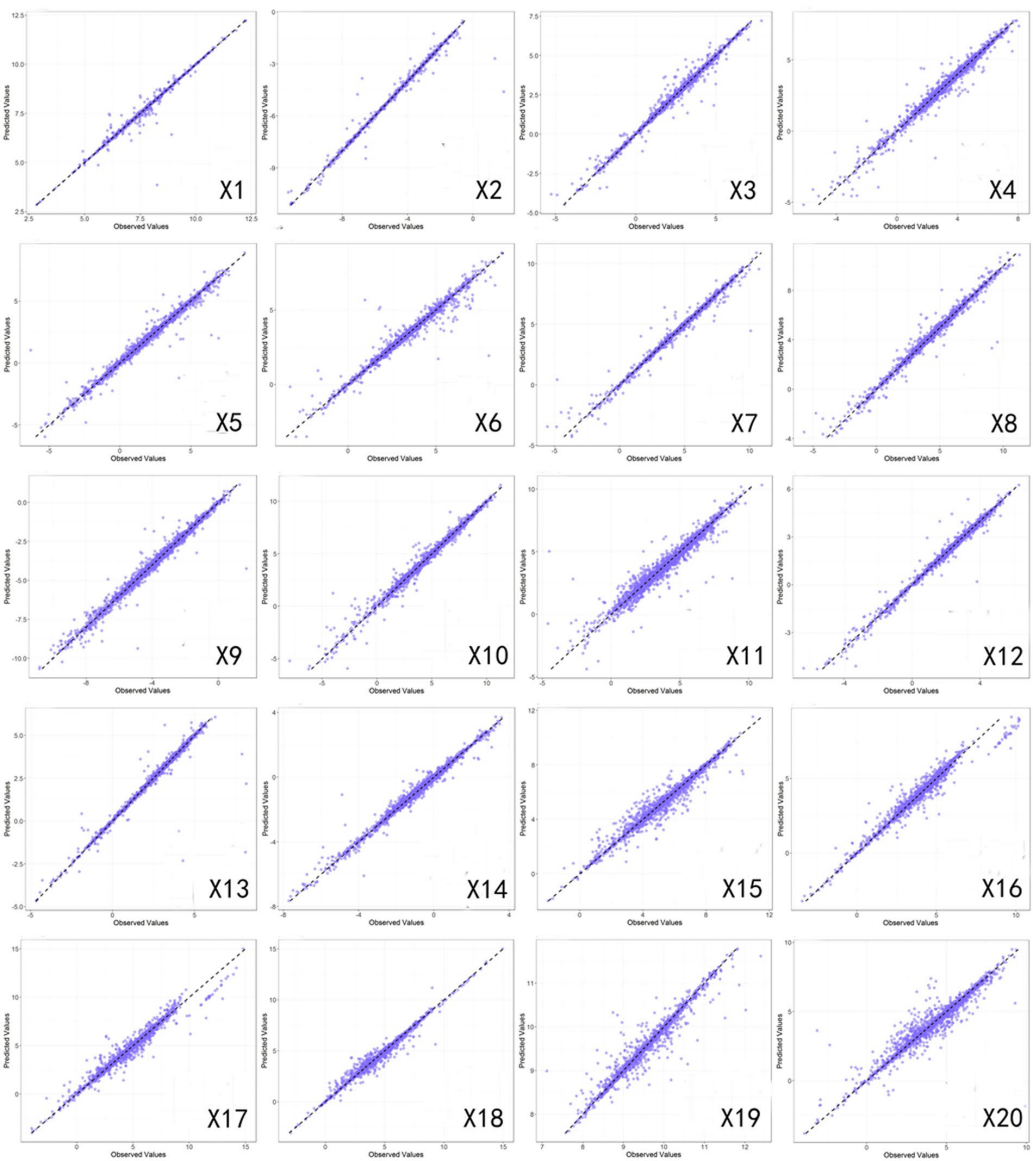
Prediction scatter diagrams of 20 variables in the 10% simulation experiment.

**Figure 4:**
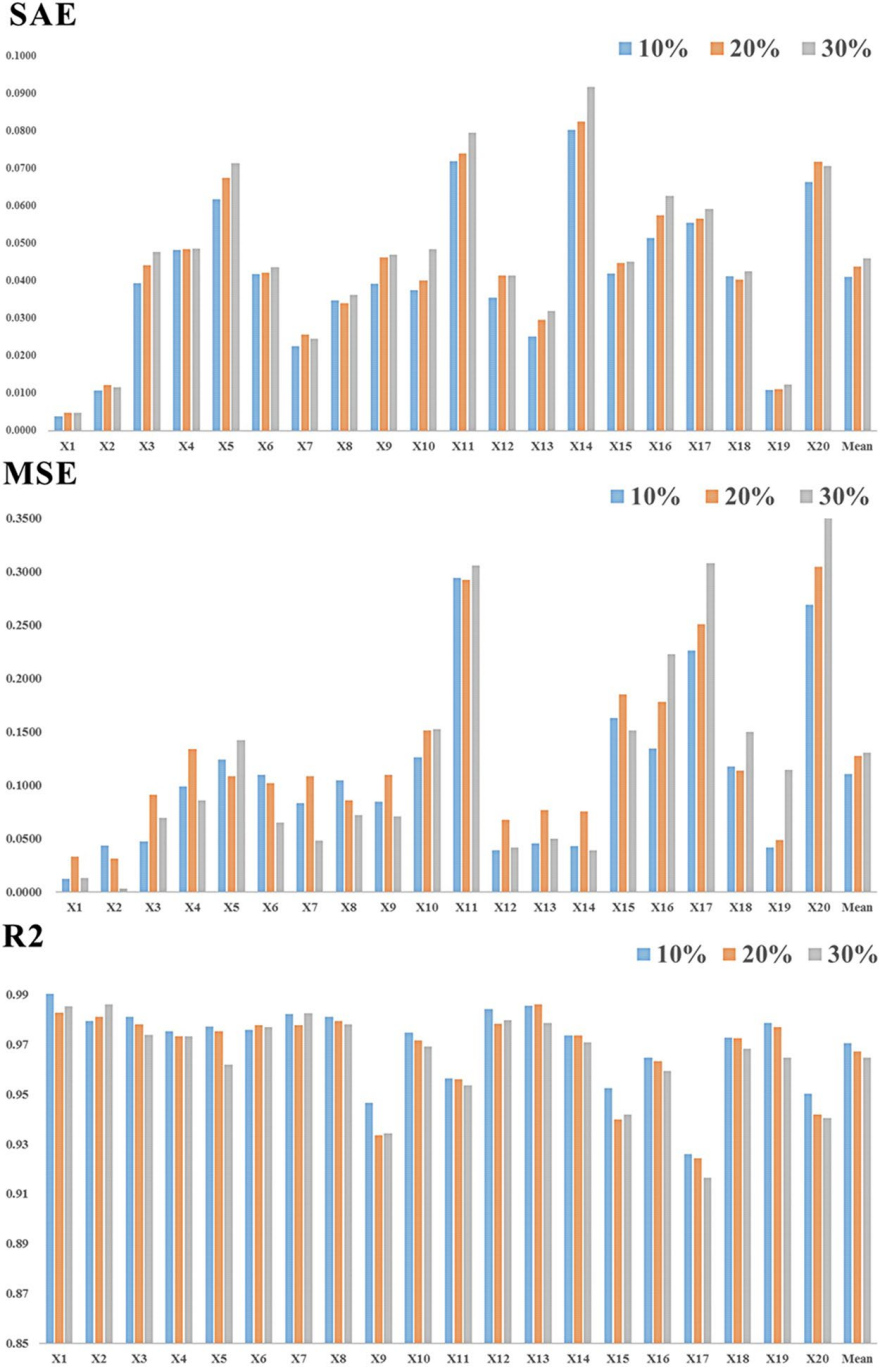
Evaluation of 20 socioeconomic variables in the 10%, 20% and 30% cross-validation simulation experiments with the PST method.

Figures 3 and 4 give the results of the cross-validation experiments with spatiotemporal Models 2 and 3. The scatter plots in Fig. 3 shows that under the 10% test set setting, the predicted values match the observed values for most variables well.

The MSE, SAE, and R^2^ consistently show that (Fig. 4) when the test set contains 10%, 20%, and 30% of all existing data in the dataset, the amount of available data for modeling in the training set decreases and the prediction error increases, but not dramatically. Since the percentage of missing data for a variable rarely goes up to 30% in our database, our models should be able to maintain an acceptable performance when applied to the database.

Furthermore, the mean SAE values of all 20 variables are less than 0.05 for all three test sets, thus indicating that the overall prediction error accuracy under these settings is less than 5%. It is noteworthy that the six variables handled by the second-step modeling, which have larger percentages of missing data, do not have considerably larger prediction errors than the 14 variables in the first-step modeling. Among all 20 variables, a total of 14 (not necessarily the first 14) have an SAE <5%. Among these 14 variables, X1, X2, and X19 are the best-estimated variables, with SAEs of approximately 1%. There are six other variables whose SAEs are between 5% and 10%, which is still acceptable.

### Spatial SAE maps

**Figure 5:**
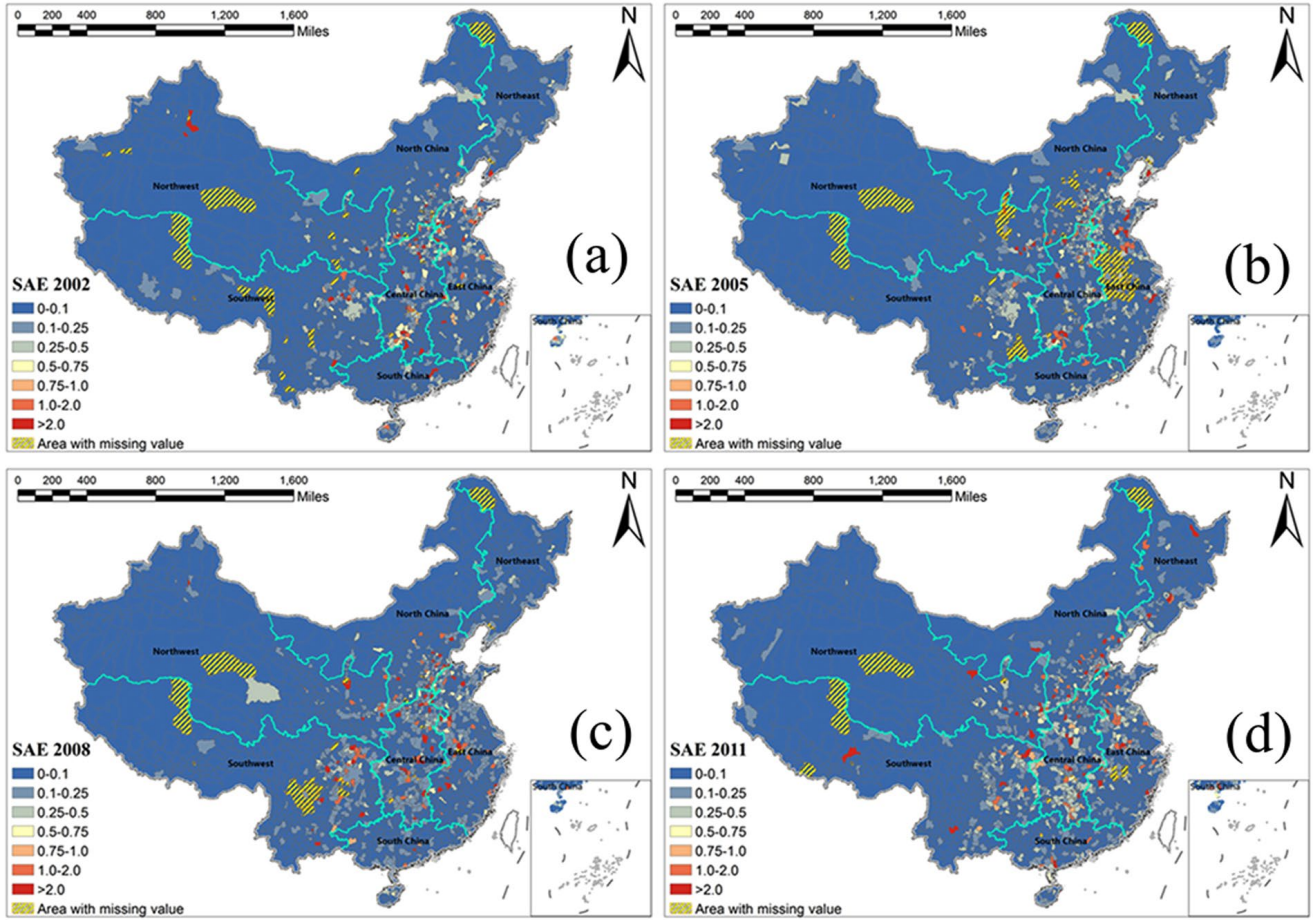
Spatial SAE maps of variable X14 in the years (**a**) 2002, (**b**) 2005, (**c**) 2008 and (**d**) 2011.

We also calcluated the localized SAE for each county to reveal the spatial variation of the uncertainty (prediction error) in the results generated by PST. As an example, Fig. 5 is a map of the local SAE for the number of hospital beds (X14) in four years. Variable X14 has the highest SAE value among the 20 variables. The map shows that most counties (blue) have a prediction error <0.1 in all four years. The regions with high-quality predictions are stable during 2002–2011, whereas the regions with relatively low- quality of predictions (red) are few and scattered. The SAE maps further illustrate the effectiveness of the applied spatiotemporal model.

### Comparison of different imputation methods

**Figure 6:**
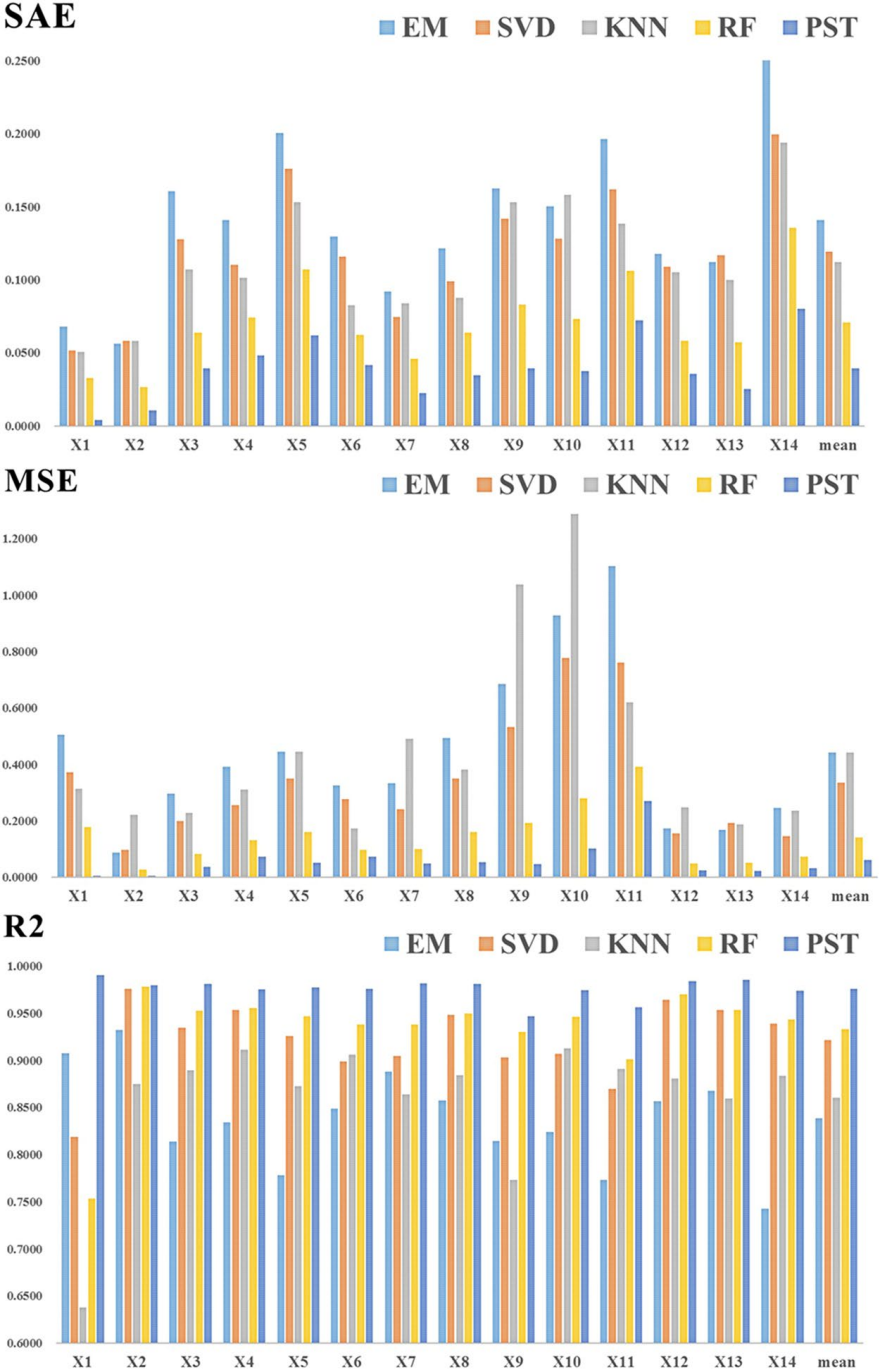
Evaluation of different imputation methods (EM, SVD, kNN, RF, and PST) for the 10% simulation dataset.

Finally, using the 10% test set, we ran cross-validation to compare the proposed PST method with four other imputation methods, including kNN, EM, SVD and RF. The comparison evaluation is still based on the SAE, MSE and R^2^. The results are shown in Fig. 6. On all three indicators, PST outperforms all other methods for all variables. For instance, the mean prediction error of PST is less than 5%, whereas that of RF is between 5% and 10%, and those of the other three methods are all greater than 10% (the top panel of Fig. 3). For the four other methods we compared, the rank from best to worst is RF, SVD, kNN and EM, and kNN and EM are almost the same. For the large-scale spatiotemporal dataset, it is useful to consider the spatial and temporal random effects as the additional information for the missing data imputation.

## Discussion

In this study, we developed a sophisticated progressive spatiotemporal (PST) method and used it to estimate the missing values in China’s county-level official socioeconomic statistics. Our estimation covers the entire country for a 10-year period and includes 20 socioeconomic variables. We developed this procedure for estimating missing values in the official statistics dataset when auxiliary samples and covariate information are not available, which is a situation that prevails in China’s socioeconomic statistics (and is also likely in other countries’ similar datasets) but would not be well addressed by previous model-based methods^5–8^. We conducted a variety of evaluations, and they consistently prove the efficacy of the proposed PST method.

PST imputes missing values using a two-step progressive modeling strategy. First, based on the understanding that socioeconomic phenomena tend to agglomerate in space and time (e.g., well-developed cities tend to promote development of nearby towns, and a county’s development tends to maintain a smooth trend during a period)^18^, we tried to derive information from those county-years that do have data by borrowing information from the spatial and temporal structures in the data and their interactivity. This first step was implemented by constructing spatiotemporal models that incorporate items of spatial autocorrelation, temporal autocorrelation, and space-time interactions, under a Bayesian hierarchical modeling framework. The BHM method is effective in taking into account non-linear spatiotemporal associations as prior information. We found that for a large country such as China, when a variable’s percentage of missing data is <15% in each year, it is possible to achieve high-quality estimation based only on the information derived from the spatial and temporal structures in the existing data. This study is a pilot study on applying this framework to the estimation of missing data in large spatiotemporal databases.

Second, when a variable has a large percentage of missing data, e.g., >85%, taking into account the imputation results of those easier-to-impute variables (variables with small percentages of missing values) can be helpful. For this purpose, we adopted a progressive strategy and implemented a two-step modeling process. That is, if some variables have been well estimated in the first step, they can be further used as covariates in the estimation for those more- difficult-to-impute variables and combined with nearby spatial and temporal information by constructing spatiotemporal multivariable regression models. This second step turned out to be effective in the estimation of the six more- difficult-to-impute variables in our study.

By comparing PST with four widely used imputation methods, including kNN, SVD, EM, and RF, we confirm that PST had a better prediction accuracy and reduced residuals compared to the other methods. The good performance of PST is greatly due to its capability to incorporate spatial and temporal autocorrelation effects, which the other four methods lack but is important for a large-scale spatiotemporal dataset. Among the other four methods, the RF method performed the best compared to the kNN, SVD, and EM imputation methods, and this result is consistent with other studies^17,45^. Especially, when a county has missing values for all variables^46^, which means that no covariates exist to estimate the target variable (covariates are fundemental to RF), PST is able to first impute those easy-to-impute variables based solely on the spatial and temporal structure information and then uses the imputation results of the easy-to-impute variables to impute those more-difficult-to-impute variables. The PST method is especially useful for the case without any additional information to use for imputation. The cross-validations also demonstrate that the performance of PST remained acceptable when the percentage of missing values went up to 30%.

The two-step PST method is not limited to the specific socioeconomic statistics variable that we have been working on, and its usefulness can be generalized. The entire procedure can be adapted and applied to the estimation of missing data for other large-scale spatiotemporal datasets. The immediate outcome of this study is a complete county-level socioeconomic dataset of China with 20 variables over a 10-year period, which should be the first of its kind. This new dataset should be of great value to multi-disciplinary research and policy-making practices.

There are some limitations to this study. This imputation method did not consider that some counties that failed to provide the required official statistics data in all ten years are also counties that are far less developed than their neighbors. Thus, assuming a smooth spatial structure when imputing missing data for these counties may result in an over-estimation. A possible solution may be to obtain more local data (unit-level) in these counties from other private sources and apply multilevel mixed models combined with the spatiotemporal models in future research. In addition, since the China National Bureau of Statistics has never publicized the standards it uses (e.g., the sampling range or the sampling method), data inconsistency has been a big concern. At this time, no other openly published county-level socioeconomic dataset is available for us to verify the data that we used in this study. Encouragingly, the results of the cross-validations indicate that even with the existence of data inconsistency, our model can still achieve a good performance and is thus valuable in imputing missing data for the official statistics. Nevertheless, data standardization is an important issue to be considered in future studies.

## Electronic supplementary material

SUPPLEMENTARY MATERIAL
