# Supplementary material for: Estimating missing values in China’s official socioeconomic statistics using progressive spatiotemporal Bayesian hierarchical modeling
Source: Sci Rep. 2018 Jul 3;8:10055. doi: 10.1038/s41598-018-28322-z (PMC6030081; doi:10.1038/s41598-018-28322-z)
Supplement: Supplementary file 1 — SUPPLEMENTARY MATERIAL [file 41598_2018_28322_MOESM1_ESM.pdf]

SUPPLEMENTARY MATERIALS

Supporting Information for the following paper

Title:

Estimating missing values in China's official socioeconomic statistics using progressive spatiotemporal Bayesian hierarchical modeling

Authors:

Chao Song*, Xiu Yang, Xun Shi*, Yanchen Bo, Jinfeng Wang

* Corresponding authors:

C.S. (songc345@163.com) and X.S. (Xun.Shi@dartmouth.edu).

Content

S1. Example of missing data situations about two portions of socioeconomic variables in the year 2009

S2. Covariate selection results for the second step of the modeling process

S3. Spatial autocorrelation test

S4. Reference

S1. Example of missing data situations about two portions of socioeconomic variables in the year 2009

Based the number of large missing percentage year, we divided the dataset into two portions for estimation with different modeling strategies. The first portion containing variables X1 to X14 is with zero large missing percentage year, and the second portion containing variables X15 to X20 is with more than one large missing percentage year.

Figure S1 presents the original missing-data situations of eight selected socioeconomic variables in the year 2009. The first four variables (X4, X5, X6, and X12) belong to the first portion, and the last four variables (X15, X16, X19, and X20) belong to the second portion. Each row bin including both blue and red ones represents one county (total: 2310 row bins or 2310 spatial county areas), and red bins represent those missing counties with missing values

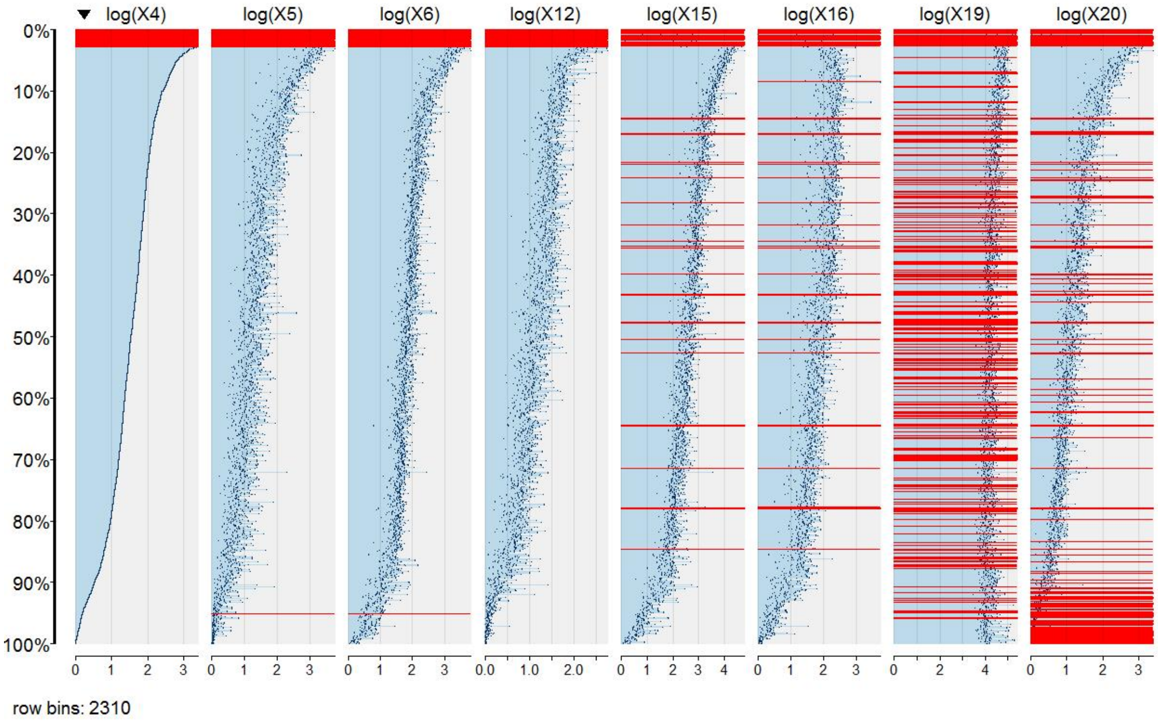

Figure S1. The original missing data situations of eight socioeconomic variables in the year 2009. Each bin represents one county (total: 2310 row bins), and red bins represent the counties with missing values

The longitudinal axis represents the index of each spatial county，and it is sorted according to the first variable (X4) in descending order. The percentage is not a cumulative percentage but represents the index of each spatial county. For example, 100% means the 2310th county, 10% means the 231st county. The horizontal axis represents the value of each variable with logarithm transformation.

For the first variable X4, among all spatial counties, the missing counties are printed in red and summarized in the front of the column. But it doesn't mean that these red bins are with high values. They are just a collection of counties with missing values for X4. Because of the one-to-one correspondence of space counties, all the other variables are drawn according to the X4 index. That's why the curve is not smooth, and the red counties with missing value are not concentrated in other seven curves except for the curve of X4.

If we draw a line around the middle 50% location in the vertical axis, we could get all values of each socioeconomic variable for the 1155th county. We find that the first four variables are with records and the last four variables are with missing values, which indicates that the missing situation is different for different socioeconomic variables in the same county. That's why we could use the other variables with records to estimate the one with missing values. Also, the socioeconomic variable X19 are with large missing data percentage (over 80%) in the year 2009. To obtain a better estimation, except for space and time information, we further use the other complete covariates from the first portion, like X4, X5, X6 and X12 in this case, as additional information to predict missing values.

For the sampled dataset of the year 2009, we can tell that some counties are missing for all variables (horizontal records with all red bins), while other areas are missing for only a few variables. Variable X19 and X20 have the large missing data problem compared to other variables in the present since they have far more red bins. We use the space-time multivariate regression model considering spatial, temporal and related covariates information to deal with these missing data problems in this paper.

S2. Covariate selection results for the second step of the modeling process

In the second step, potential covariates in a Bayesian ecological model were selected from the newly imputed and complete fourteen variables, covX1 to covX14, estimated by the first-step models. One important task here is to select the candidate variables to build a multivariable regression model for second-step missing data estimation. We calculate the variance inflation factor (VIF) and tolerance for each candidate variable to assess the multicollinearity. Then, we use the forward stepwise regression method to exclude the variables without statistical significance.

We considered the VIF < 5, tolerance > 0.1 and significance (sig) < 0.05 as screening standards[1] for variable selection to avoid multicollinearity[2] and statistically insignificant factors in SPSS 22.0 software[3].

First, we calculated the VIFs and tolerances of all 14 covariates, covX1 to covX14; see Table S1. Tolerance, such as VIF, is also a useful index to evaluate multicollinearity. Note that the covariates used here were those of the complete filled dataset after prediction by the first-step models. We removed five variables with higher VIF and lower tolerance values according to the screening criteria above.

Table S1. Multicollinearity evaluation results

Covariates	VIF	Tolerance
covX1	1.113	0.898
covX2	1.183	0.845
covX3	6.034	0.166
covX4	8.941	0.112
covX5	6.705	0.149
covX6	3.304	0.303
covX7	3.222	0.310
covX8	3.419	0.293
covX9	1.945	0.514
covX10	2.741	0.365
covX11	2.430	0.411
covX12	55.338	0.018
covX13	42.724	0.023
covX14	1.837	0.544

Then, we built the multiple forward stepwise regression models to exclude the variables without statistical significance (sig >0.05) for each dependent socio-economic variable in the second modeling stage. Here, we list the final selection results of the dependent variable X15; see Table S2. Beta is the standardized regression coefficient that is estimated in SPSS. Indicator t is the t-test result, which is used to calculate the sig value. We find that for the dependent variable X15, seven covariates without covX7 (sig=0.332) and covX8 (sig=0.399) are used for the second-step Bayesian spatiotemporal ecological modeling. Table S3 summarizes the covariate selection results of all dependent socio-economic variables in the second modeling step.

Table S2. Multiple and forward stepwise regression results of X15

Model	Covariate	VIF	Tolerance	Beta	t	sig
Multiple regression	covX1	1.129	0.886	0.126	14.616	0
	covX2	1.038	0.963	0.037	4.501	0
	covX6	2.220	0.450	0.048	3.984	0
	covX7	2.909	0.344	0.013	0.970	0.332
	covX8	2.777	0.360	0.011	0.843	0.399
	covX9	1.983	0.504	-0.102	-8.958	0
	covX10	2.393	0.418	0.125	9.982	0
	covX11	2.283	0.438	0.087	7.095	0
	covX14	1.611	0.621	0.026	2.532	0.011
Forward stepwise regression	covX1	1.109	0.902	0.124	14.535	0
	covX2	1.029	0.971	0.038	4.662	0
	covX6	2.079	0.481	0.053	4.530	0
	covX9	1.652	0.605	-0.095	-9.108	0
	covX10	2.255	0.443	0.130	10.675	0
	covX11	2.226	0.449	0.090	7.439	0
	covX14	1.448	0.691	0.031	3.204	0.001

Table S3. Summary of covariates selection results in the second modeling step

Dependent variable	Covariates number	Selected covariates (covX)
X15	9	1 2 6 7 8 9 10 11 14
X16	7	1 6 8 9 10 11 14
X17	6	1 6 8 9 10 11
X18	7	1 6 8 9 10 11 14
X19	7	1 2 6 9 10 11 14
X20	8	1 2 6 7 8 9 11 14

S3. Spatial autocorrelation test

Spatial autocorrelation refers to the potential interdependence between the observed data (areas) in the same distribution zone[4] and is described as the structured spatial effect in this study[5]. We performed a full test for each socio-economic variable in each year to test whether it is necessary to introduce this structured spatial effect (significant or insignificant spatial autocorrelation) by using a mature test tool named "Spatial Autocorrelation" in ArcGIS 10.2[6]. This tool helps us understand the degree to which one area is similar to other nearby areas[7]. Moran's Index (Moran's I) is widely used to measure spatial autocorrelation and is calculated as[8]:

$$I = \frac{\sum_{i=1}^{n}\sum_{j=1}^{n} w_{ij}\left(x_i - \bar{x}\right)\left(x_j - \bar{x}\right)}{S^2 \sum_{i=1}^{n}\sum_{j=1}^{n} w_{ij}} \ , \ i \neq j$$

$$S^2 = \frac{1}{n}\sum_{i=1}^{n}\left(x_i - \bar{x}\right)^2 \ , \quad \bar{x} = \frac{1}{n}\sum_{i=1}^{n} x_i$$

where n is the sample size, x_i and x_j represent the attribute values of areas i and j, and w_{ij} represents the weighting of spatial relationships, where if areas i and j are neighbors, then w_{ij}=1, otherwise w_{ij}=0. In the case of the Spatial Autocorrelation tool, the null hypothesis states that "there is no spatial clustering of the values associated with the geographic features in the study area." When the p-value is small, and the absolute value of the Z-score is large enough that it falls outside of the desired confidence level, the null hypothesis can be rejected [9]. If the value of Moran's I is greater than 0, the set of features exhibits a clustered pattern (structured spatial effect). If the value is less than 0, the set of features exhibits a dispersed pattern.

We list the p-value and Z-score threshold standards of different confidence intervals in Table S4 and further give an output example of one variable by using the Spatial Autocorrelation tool in ArcGIS to explain the results; see Figure S2.

Table S4. Moran's I test standards

Z-score	p-value	Confidence
<-1.65 or >+1.65	<0.10	90%
<-1.96 or >+1.69	<0.05	95%
<-2.58 or >+2.58	<0.01	99%

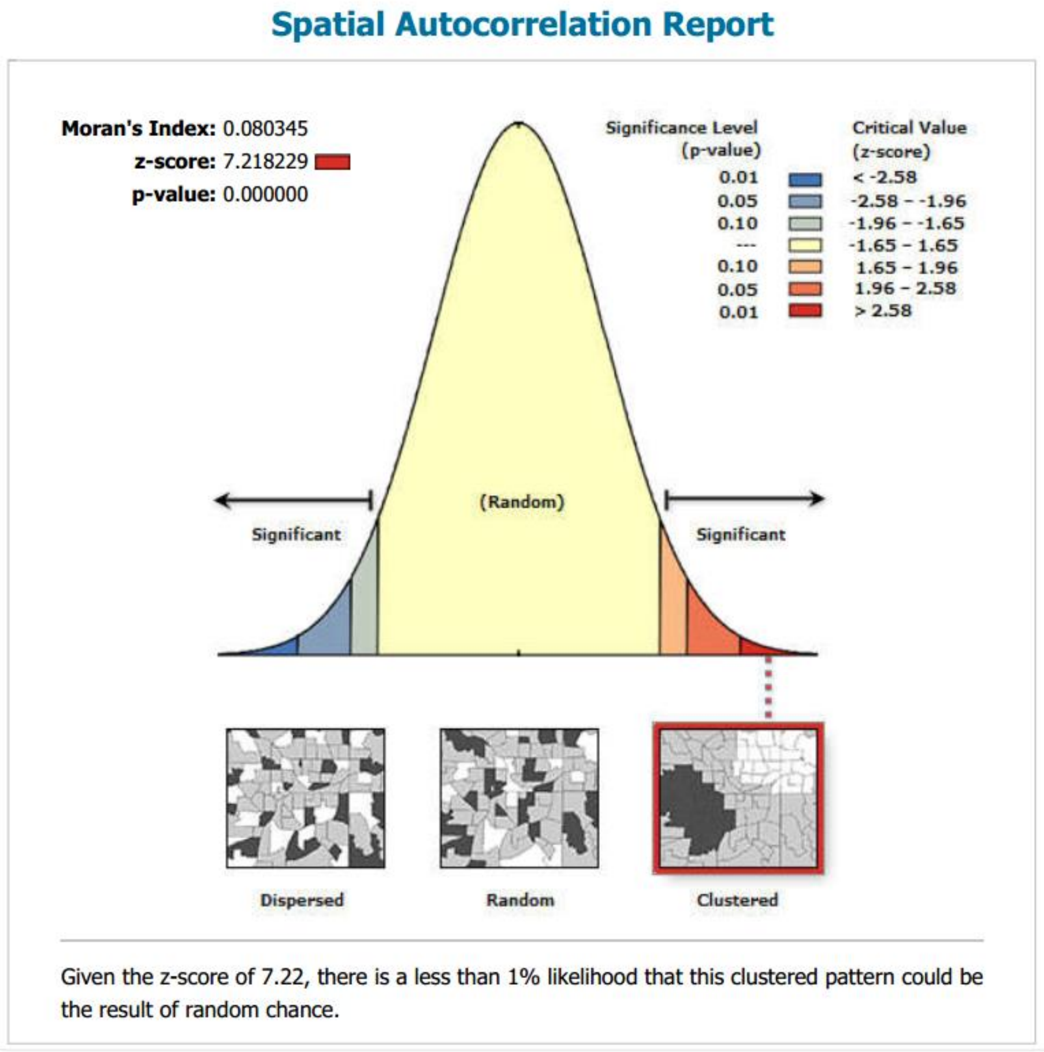

Figure S2. Spatial autocorrelation report of variable X3 in the year 2002

From Table S4 and Figure S2, we can see that Z-score is the most important indicator for interpreting whether the target variable is clustered or dispersed. Under the 99% confidence condition (p-value<0.01), if the Z-score is larger than 2.58, then the variable is spatially positively correlated and clustered, which indicates that it is reasonable to consider spatial autocorrelation to build a spatial model. In this case, the

Z-score is 58.56, which is much larger than 2.58; this shows that this variable rejects the null hypothesis and has the possibility (the possibility is greater than 99%) of significant clustering and positive spatial correlation.

From this point of view, we further summarize the Z-score values of all twenty socio-economic variables for each year; see Table S5. We find that all the Z-score values are positive and greater than 2.58, which indicates that it is necessary to consider spatial autocorrelation effects for all socioeconomic variables in spatiotemporal models in this case. The Moran's I test also showed that the spatial structure based on adjacency method was able to represent the significant spatial autocorrelation relationships for all variables in each year.

Table S5. Summary of Z-score values in spatial autocorrelation tests for all 20 variables

Variable	2002	2003	2004	2005	2006	2007	2008	2009	2010	2011
X1	42.19	37.31	37.39	42.97	42.84	40.03	40.67	42.00	42.23	42.05
X2	25.17	24.84	24.70	22.79	21.87	22.24	22.68	22.82	22.91	21.76
X3	7.22	6.96	7.06	6.88	7.50	7.70	7.52	7.68	7.76	7.06
X4	14.14	7.50	13.97	14.05	13.73	13.59	12.88	12.89	12.50	12.97
X5	9.49	9.20	8.58	10.24	9.85	9.53	9.67	10.23	10.81	11.05
X6	9.15	9.14	8.57	8.69	8.19	9.38	9.58	9.99	10.27	10.54
X7	9.20	9.66	9.11	9.49	9.18	9.54	9.60	9.73	8.43	9.61
X8	7.51	7.88	7.54	8.27	8.38	8.52	8.50	8.90	10.81	9.08
X9	11.61	21.01	21.79	21.94	21.98	23.43	21.98	24.97	27.57	27.44
X10	15.21	15.53	16.35	18.00	16.09	16.82	16.51	18.37	18.87	18.97
X11	8.13	10.48	10.96	14.19	12.91	12.96	13.46	13.79	14.44	15.34
X12	26.42	29.16	29.13	27.40	26.21	26.88	26.09	25.77	24.90	22.96
X13	33.19	31.79	30.86	28.22	27.02	26.95	24.86	25.53	24.74	23.46
X14	10.81	10.74	10.32	10.76	10.53	10.40	10.36	9.61	9.78	9.82
X15	14.00	14.26	12.88	11.54	11.00	13.45	11.23	13.69	14.16	\
X16	\	\	18.26	24.46	23.29	42.15	24.58	42.64	44.15	2.98
X17	\	\	13.98	13.12	12.34	16.05	12.67	17.09	16.79	7.64
X18	\	\	10.46	8.79	8.49	8.91	8.47	9.84	10.35	\
X19	\	\	\	27.72	27.35	45.39	19.66	43.61	40.09	\
X20	10.94	10.92	10.13	10.49	10.25	11.45	9.78	11.15	11.62	\

Note: "\" means there exists a large missing percentage of missing data in that year

S4. Reference

1 Bo, Y., Song, C., Wang, J. & Li, X. Using an autologistic regression model to identify spatial risk factors and spatial risk patterns of hand, foot and mouth disease (HFMD) in Mainland China. *BMC Public Health* **14**, 358, doi:10.1186/1471-2458-14-358 (2014).

2 Vatcheva, K., Lee, M., McCormick, J. & Rahbar, M. Multicollinearity in regression analyses conducted in epidemiologic studies. *Epidemiology (Sunnyvale, Calif.)* **6** (2016).

3 Cronk, B. *How to use SPSS®: A step-by-step guide to analysis and interpretation*. (Routledge, 2017).

4 Ord, J. & Getis, A. Local spatial autocorrelation statistics: distributional issues and an application. *Geographical analysis* **27**, 286-306 (1995).

5 Blangiardo, M., Cameletti, M., Baio, G. & Rue, H. Spatial and spatio-temporal models with R-INLA. *Spatial and spatio-temporal epidemiology* **7**, 39-55 (2013).

6 Wong, W. & Lee, J. *Statistical analysis of geographic information with ArcView GIS and ArcGIS*. (Wiley, 2005).

7 Griffith, D. Spatial autocorrelation. *A Primer (Washington, DC, Association of American Geographers)* (1987).

8 Goodchild, M. *Spatial autocorrelation*. Vol. 47 (Geo Books, 1986).

9 Getis, A. & Ord, J. The analysis of spatial association by use of distance statistics. *Geographical analysis* **24**, 189-206 (1992).
